# Analysis of factors related to anesthetic management affecting acute kidney injury occurring within 72 h after esophagectomy for esophageal cancer: a historical cohort study

**DOI:** 10.1186/s40981-024-00756-7

**Published:** 2024-11-28

**Authors:** Seiji Ishikawa, Junko Hirashima, Makiko Hiroyama, Shojiro Ozato, Masayuki Watanabe, Katsuyuki Terajima

**Affiliations:** 1 Department of Anesthesiology, The Cancer Institute Hospital of Japanese Foundation for Cancer Research 3-8-31, Ariake, Koto-ku, Tokyo 135-8550 Japan; 2 Department of Anesthesiology and Pain Medicine, Faculty of Medicine, Juntendo University, 2-1-1, Hongo, Bunkyo-ku, Tokyo 113-8421 Japan; 3 Department of Gastroenterological Surgery, The Cancer Institute Hospital of Japanese Foundation for Cancer Research, 3-8-31, Ariake, Koto-ku, Tokyo 135-8550 Japan

**Keywords:** Acute kidney injury, Epidural anesthesia, Esophageal cancer, Esophagectomy, Renal protection, Postoperative complication, Risk factors

## Abstract

**Background:**

The effects of factors related to anesthetic management, including anesthesia methods and infusion volume, on acute kidney injury (AKI) after esophagectomy have not been thoroughly investigated.

**Methods:**

A historical cohort study of patients who underwent esophagectomy between January 2008 and December 2022 was conducted. AKI was defined according to the Kidney Disease Improving Global Outcomes creatinine criteria within 72 h after esophagectomy. Logistic regression was used to model the association between perioperative factors, including factors related to anesthetic management, and postoperative AKI.

**Results:**

Of 1005 patients, 48 patients (4.8%) had AKI (40 stage 1 and 8 stage 2). AKI patients were older (67.8 vs. 65.0 years, *P* = 0.046) and more likely to have hypertension (72.9 vs. 37.9%, *P* < 0.001), chronic kidney disease (39.6 vs. 14.3%, *P* < 0.0001), red blood cell (RBC) transfusions (12.5 vs. 3.4%, *P* = 0.0085), and longer duration of anesthesia (518 vs. 490 min, *P* = 0.0058) than non-AKI patients. AKI patients were less likely to have epidural anesthesia (72.9 vs. 91.5%, *P* < 0.001). The distribution of inhaled anesthetics chosen was not significantly different between AKI and non-AKI patients. On multivariable logistic regression analysis, AKI was associated with the Brinkman index (per 100 units, odds ratio (OR) = 1.06), hypertension (OR = 3.39), chronic kidney disease (OR = 2.58), duration of anesthesia (per 10 min, OR = 1.03), epidural anesthesia (OR = 0.35) and RBC transfusion (OR = 3.27).

**Conclusions:**

Except for epidural anesthesia, no significant association was found between AKI and factors related to anesthetic management. Epidural anesthesia may protect against early postoperative AKI in patients undergoing esophagectomy.

## Background

Esophagectomy is a complex surgical procedure involving the neck, chest, and abdomen that has significant mortality and morbidity. While the use of minimally invasive esophagectomy (MIE), which includes thoracoscopic, laparoscopic, mediastinoscopic, and robotic surgery, for esophageal cancer seems to be increasing globally [1], postoperative mortality and total morbidities remain high, ranging from 1.1% to 2.8% and 40.7% to 42.9%, respectively [2, 3]. Therefore, measures to prevent various postoperative complications may be helpful in improving the outcomes of patients undergoing esophagectomy.

Acute kidney injury (AKI) is one of the postoperative complications that has been shown to worsen both short-term [4, 5] and long-term outcomes [6, 7] in a variety of surgical procedures. Because anesthetic management, such as the anesthesia method, and the amount of fluid administered during surgery, may affect a patient’s inflammatory response and fluid status during and after surgery, early postoperative AKI appears to be strongly related to anesthetic management. Therefore, if the risk of early postoperative AKI can be reduced by improving anesthetic management, this may help improve the outcomes of patients undergoing esophagectomy. However, to the best of our knowledge, few studies have analyzed the risk of AKI after esophagectomy, taking into account anesthesia-related factors. For example, there has been only one study examining factors, including epidural anesthesia, associated with early postoperative AKI [8].

The goal of anesthesiologists is to improve patients’ outcomes by providing the best possible anesthetic management in patients undergoing esophagectomy. To achieve this goal, the present study focused on AKI in the early postoperative period and attempted to identify anesthesia-related factors that may help us improve anesthetic management. The objectives of the present study were threefold: (1) to calculate the incidence of AKI within 72 h after esophagectomy; (2) to identify anesthesia-related factors related to early postoperative AKI; and (3) to investigate the effects of AKI on outcomes.

## Methods

This study was approved by the Institutional Review Board of The Cancer Institute Hospital of Japanese Foundation for Cancer Research (No. 1682, June 15, 2023), and the need for informed consent was waived. This report is based on our single-center, historical cohort study of patients who underwent esophagectomy and reconstruction and is in keeping with the Strengthening the Reporting of Observational Studies in Epidemiology (STROBE) guidelines [9].

### Patient inclusion and data collection

A historical cohort study of patients who underwent esophagectomy for esophageal cancer between January 2008 and December 2022 was conducted. Patients who received hemodialysis preoperatively were excluded. Patients who underwent esophagectomy without thoracotomy or thoracoscopy (e.g., transhiatal esophagectomy, mediastinoscopic esophagectomy) were excluded because our interest was in the protective effects that MIE might have on the kidneys compared with open thoracotomy, as well as factors related to anesthetic management. Patients who underwent two-stage esophagectomy were also excluded because two-stage surgery may be less invasive than one-stage surgery.

Patient data, surgical data, and anesthetic data, as well as laboratory data, were manually abstracted from the patients’ electronic charts. Data collected included: age, sex, height, body weight, body mass index (BMI), American Society of Anesthesiologists physical status (ASA-PS), history of cigarette smoking (Brinkman index), history of hypertension, diabetes mellitus, asthma, chronic obstructive pulmonary disease, heart disease, peripheral vascular disease, and chronic kidney disease (CKD), history of chemotherapy and radiotherapy, cancer histology, tumor location and preoperative cancer stage. The Union for International Cancer Control (UICC) Tumor Node Metastasis (TNM) Classification of Malignant Tumors, 8th edition, was used for staging esophageal cancer. Preoperative medications examined were: angiotensin-converting enzyme (ACE) inhibitors, angiotensin II receptor blockers (ARBs), steroids, non-steroidal anti-inflammatory drugs (NSAIDs), statins, and diuretics. Preoperative laboratory values including hemoglobin, platelet, albumin, aspartate aminotransferase, alanine aminotransferase, total bilirubin, and serum creatinine (sCr) levels were obtained from the computerized medical records. The preoperative glomerular filtration rate (GFR) was estimated from the formula developed for Japanese adult patients and adjusted for each 1.73 m^2^ of body surface area: estimated GFR (eGFR) (mL/min/1.73 m^2^) = 194 × sCr (mg/dL)^(−1.094)^ x Age (y)^(−0.287)^ × 0.739 (if female) [10]. CKD was defined as a baseline eGFR ≤ 60 mL/min/1.73 m^2^. Surgical and anesthetic factors recorded were: MIE or open thoracotomy, organ used for reconstruction, route of reconstruction, maintenance anesthetic agent (inhalational or total intravenous anesthesia (TIVA)), thoracic epidural anesthesia, intraoperative administration of catecholamines (dopamine, dobutamine, norepinephrine, and epinephrine), ventilatory management (one-lung or two-lung ventilation), duration of surgery and anesthesia, volume and type of intraoperative fluids (crystalloid or hydroxyethyl starch) and albumin administered. Intraoperative transfusions of red blood cells (RBCs), fresh frozen plasma, or platelets, urine output, and estimated blood loss were recorded. One unit of each blood component was derived from 200 mL of whole blood.

### Surgery and management of anesthesia

The anesthetic management method of choice for esophageal surgery, whether MIE or open thoracotomy, was basically a combination of general anesthesia and epidural anesthesia at our institution. The choice of maintenance anesthetic agent and analgesic drugs was left to the discretion of the anesthesiologists. Patient-controlled analgesia was routinely used epidurally for postoperative analgesia. If epidural anesthesia was not used for some reason, intravenous patient-controlled analgesia was used instead. MIE for esophageal cancer in the prone position was introduced in 2010 in our institution. Although one-lung ventilation with a double-lumen tube or a bronchial blocker was used when it was first implemented, ventilatory management was shifted from one-lung ventilation to two-lung ventilation in 2013 in patients who underwent MIE. At the end of the surgery, patients were routinely extubated in the operating room if there were no safety issues, such as a high risk of postoperative respiratory failure.

### Definitions and outcomes

This study investigated the incidence of and risk factors for AKI after esophagectomy. Postoperative AKI was considered present if the postoperative sCr concentration reached ≥ 1.5 times the preoperative (baseline) sCr within 72 h after surgery or if there was an increase in sCr ≥ 0.3 mg/dL within 48 h during the 72-h observational period after surgery according to the Kidney Disease Improving Global Outcomes (KDIGO) creatinine criteria [11]. AKI in the early stage of the postoperative period was selected because it may be related to anesthetic management, and clarifying risk factors for early AKI after esophagectomy may facilitate improvements in anesthetic management. AKI that occurs in the later stage of the postoperative period was not included because it may be related to surgical complications (e.g., sepsis) rather than anesthetic management. The KDIGO urine output criteria for AKI were not used in the present study because of the possible lack of completeness and accuracy of both the measurement and recording of postoperative urine output.

### Sample size estimation

Sample size was not estimated because the entire accessible cohort was included in this study.

### Statistical analysis

Continuous variables are summarized as median (25th, 75th percentiles) or mean (standard deviation) values. Categorical variables are summarized as frequencies (percentages). Normally and non-normally distributed continuous data were analyzed using Student’s two-sample *t*-test and the Mann–Whitney *U* test, respectively. Categorical data were analyzed using Fisher’s exact test.

Multivariable logistic regression was used to model the relationship between postoperative AKI developing within 72 h and perioperative risk factors. To identify perioperative risk factors, exploratory data analysis was first performed using univariate comparisons. Then, the model included all covariates of clinical importance (age and sex), factors related to anesthetic management of interest (anesthetic agent (inhalational vs TIVA), epidural anesthesia, total intravenous fluid volume, and hydroxyethyl starch), and MIE or open thoracotomy regardless of statistical significance, and all covariates with associations on exploratory analysis (*P* < 0.1). To avoid multicollinearity, variables with high variance inflation factors (VIFs) (> 5) and/or with high correlation coefficients (*r* > 0.8) were not included, and the analyses were performed by backward stepwise selection methods. Data are presented as odds ratios with 95% confidence intervals. To confirm the relationship between AKI and anesthesia-related factors obtained by multivariable logistic regression analysis as a sensitivity analysis, factors other than anesthesia-related factors that had an independent relationship with AKI were excluded one by one, and multivariable logistic regression analysis was performed.

All tests were 2-sided, and a* P* value < 0.05 was considered significant. All statistical analyses were conducted using EZR (Saitama Medical Center, Jichi Medical University, Saitama, Japan), which is a graphical user interface for R (The R foundation for Statistical Computing, Vienna, Austria) [12].

## Results

A total of 1035 patients who underwent esophagectomy between January 2008 and December 2022 were identified for this study. None of the patients received dialysis preoperatively. After excluding 6 patients who underwent transhiatal esophagectomy, 13 patients who underwent mediastinoscopic esophagectomy, and 11 patients who underwent two-stage esophagectomy, 1005 patients were included in the final analysis. AKI was diagnosed in 48 patients (4.8%) within 72 h after surgery, of which 40 were stage 1, and 8 were stage 2. The incidence of AKI was 6.9% after open thoracotomy and 3.9% after MIE, with no significant difference (*P* = 0.052) between them.

Patients who developed AKI were significantly older, more likely to be male, and have higher ASA-PS and Brinkman index than non-AKI patients. AKI patients were more likely than non-AKI patients to have a history of hypertension, diabetes mellitus, heart disease, and CKD, and take ARBs. Hemoglobin, serum albumin concentration, and eGFR were lower, and sCr was higher in AKI patients than non-AKI patients (Table 1).

**Table 1:** Patients’ preoperative characteristics

	AKI (*n* = 48)	Non-AKI (*n* = 957)	*P *value
Demographic factors			
Age, y (SD)	67.8 (6.7)	65.0 (9.5)	0.046
Female, N (%)	3 (6.2)	183 (19.1)	0.022
Height, cm (SD)	163.7 (7.3)	164.9 (7.8)	0.31
Weight, kg (SD)	60.9 (12.1)	59.5 (11.1)	0.40
BMI, kg/m^2^ (SD)	22.6 (3.6)	21.8 (3.2)	0.092
ASA physical status, N (%)			0.0054
1	1 (2.1)	147 (15.4)	
2	42 (87.5)	764 (79.8)	
3	5 (10.4)	46 (4.8)	
4	0 (0.0)	0 (0.0)	
5	0 (0.0)	0 (0.0)	
Brinkman index (IQR)	710 (425-1015)	528 (140-880)	0.031
Chemotherapy, N (%)	26 (54.2)	517 (54.1)	1
Radiotherapy, N (%)	4 (8.3)	60 (6.3)	0.54
Esophageal cancer			
Cancer histology, N (%)			0.28
Squamous cell, N (%)	38 (79.2)	821 (85.8)	
Adenocarcinoma, N (%)	9 (18.8)	118 (12.3)	
Other, N (%)	1 (2.1)	18 (1.9)	
Tumor location			0.33
Upper, N (%)	10 (20.8)	142 (14.8)	
Middle, N (%)	22 (45.8)	410 (42.8)	
Lower, N (%)	16 (33.3)	405 (42.3)	
Preoperative cancer stage, N (%) ^a^			0.90
0	0 (0.0)	6 (0.6)	
I	19 (39.6)	345 (36.1)	
II	12 (25.0)	238 (24.9)	
III	13 (27.1)	302 (31.6)	
IV	4 (8.3)	66 (6.9)	
Comorbidity, N (%)			
Hypertension	35 (72.9)	362 (37.9)	< 0.001
Diabetes mellitus	11 (22.9)	103 (10.8)	0.017
Asthma	0 (0.0)	34 (3.6)	0.40
COPD	8 (16.7)	124 (13.0)	0.51
Heart disease	14 (29.2)	112 (11.7)	0.0013
Peripheral vascular disease	3 (6.2)	15 (1.6)	0.051
Chronic kidney disease	19 (39.6)	137 (14.3)	< 0.0001
Perioperative medication, N (%)			
ACE inhibitor	1 (2.1)	7 (0.7)	0.32
ARB	14 (29.2)	149 (15.6)	0.025
Steroid	0 (0.0)	8 (0.8)	1
NSAID	1 (2.1)	53 (5.5)	0.51
Statin	6 (12.5)	81 (8.5)	0.30
Diuretic	3 (6.2)	21 (2.2)	0.10
Laboratory test			
Hemoglobin, g/dL (SD)	12.0 (1.6)	12.5 (1.6)	0.041
Platelets, x 10^3^/μL (SD)	215 (81)	232 (72)	0.10
Albumin, g/dL (SD)	3.9 (0.4)	4.0 (0.4)	0.028
AST, IU/L (SD)	20.8 (6.9)	22.0 (10.6)	0.42
ALT, IU/L (IQR)	17.9 (9.7)	18.5 (16.1)	0.80
Total bilirubin mg/dL (SD)	0.48 (0.21)	0.48 (0.22)	0.98
Creatinine, mg/dL (SD)	0.97 (0.31)	0.77 (0.19)	< 0.0001
eGFR, mL/min/1.73 m^2^ (SD)	65.0 (19.6)	77.9 (18.3)	< 0.0001

*AKI* acute kidney injury, *SD* standard deviation, *BMI* body mass index, *ASA* American Society of Anesthesiologists, *IQR* interquartile range, *COPD* chronic obstructive pulmonary disease, *ACE* angiotensin-converting enzyme, *ARB* angiotensin II receptor blocker, *NSAID* non-steroidal anti-inflammatory drug, *AST* aspartate aminotransferase, *ALT* alanine aminotransferase, *eGFR* estimated glomerular filtration rate

^a^The Union for International Cancer Control (UICC) Tumor Node Metastasis (TNM) Classification of Malignant Tumors, 8th edition, was used for staging esophageal cancer

There were significant differences in which reconstruction route was chosen between AKI and non-AKI patients. Duration of surgery and anesthesia were significantly longer in AKI patients than in non-AKI patients. Epidural anesthesia was more likely chosen in non-AKI patients than in AKI patients, though no difference was found in the anesthetic agents (inhalational vs. TIVA) between them. Epidural anesthesia was not performed in 94 patients. The reasons for not performing epidural anesthesia included preoperative drug administration (anticoagulants, antiplatelet drugs, etc.) in 42 patients, planned postoperative anticoagulant therapy in 22 patients, unknown in 16 patients, preoperative abnormal test results due to comorbid diseases (coagulation function, etc.) in 12 patients, preexisting neurological disorder in 1 patient, and patient non-consent in 1 patient.

There were no significant differences in the amount of crystalloid, hydroxyethyl starch, or albumin as well as total of intravenous fluid, administered between AKI and non-AKI patients. The amount of blood loss during surgery was higher in AKI patients than in non-AKI patients, and the frequency of RBC transfusions was higher in AKI patients than in non-AKI patients. Only 10 and 1 patients received fresh frozen plasma and platelet during anesthesia, respectively. There were significant differences between the groups in intraoperative fluid balance and intraoperative blood balance. The proportion of patients who received dopamine was significantly higher in the AKI group than in the non-AKI group (Table 2). No patients received dobutamine or epinephrine.

**Table 2:** Perioperative variables

	AKI (*n* = 48)	Non-AKI (*n* = 957)	*P *value
Surgery			
Minimally invasive esophagectomy, N (%)	27 (56.2)	673 (70.3)	0.052
Reconstructedorgans, N (%)			1
Stomach	47 (97.9)	920 (96.1)	
Others	1 (2.1)	37 (3.9)	
Reconstruction route, N(%)			0.0065
Retrosternal	22 (45.8)	519 (54.2)	
Intrathoracic	14 (29.2)	284 (29.7)	
Posterior mediastinum	6 (12.5)	133 (13.9)	
Others	6 (12.5)	21 (2.2)	
Duration of surgery, min (IQR)	604 (551-720)	565 (502-642)	0.0028
Anesthesia			
Anesthetic agent, N (%)			1
Inhalational	47 (97.9)	918 (95.9)	
TIVA	1 (2.1)	39 (4.1)	
Epidural, N(%)	35 (72.9)	876 (91.5)	< 0.001
Ventilatory management			0.043
One-lung ventilation	23 (47.9)	320 (33.4)	
Two-lung ventilation	25 (52.1)	637 (66.6)	
Total intravenous fluid, mL(IQR)	4665 (3725-5935)	4300 (3600-5200)	0.058
Crystalloid, mL(IQR)	3650 (3000-4553)	3300 (2700-4170)	0.097
Hydroxyethyl starch, mL(IQR)	1000 (500-1000)	1000 (500-1000)	0.94
Albumin, mL(IQR)	0 (0-475)	0 (0-250)	0.10
Estimated blood loss, mL(IQR)	280 (168-543)	140 (70-315)	< 0.001
Urine volume, ml (IQR)	590 (408-974)	630 (420-1000)	0.60
RBC transfusion, N (%)	6 (12.5)	33 (3.4)	0.0085
Intraoperative fluid balance, mL (IQR)	3955 (3233-4883)	3520 (2980-4270)	0.015
Intraoperative blood balance, mL (IQR)	-250 (-433--120)	-130 (-300--70)	<0.001
Administration of catecholamines during anesthesia			
Dopamine, N (%)	11 (22.9)	109 (11.4)	0.023
Norepinephrine, N (%)	3 (6.2)	41 (4.3)	0.46
Duration of anesthesia, min (IQR)	518 (479-641)	490 (426-567)	0.0058

*AKI* acute kidney injury, *IQR* interquartile range, *TIVA* total intravenous anesthesia, *RBC* red blood cell

The multivariable model for developing AKI was adjusted for age, sex, BMI, Brinkman index, hypertension, diabetes mellitus, heart disease, CKD, peripheral vascular disease, ARBs, preoperative hemoglobin concentration, preoperative albumin concentration, dopamine, duration of anesthesia, epidural anesthesia, anesthetic agent, MIE, reconstruction route, total intravenous fluid volume, hydroxyethyl starch, estimated blood loss, and RBC transfusion. Duration of surgery was not included in the model since it was highly correlated with the duration of anesthesia (*r* = 0.95). Intraoperative fluid balance and intraoperative blood balance were not included in the multivariable logistic regression model because their VIF values were > 5. ASA-PS was not included in the multivariable model because it is related to other variables such as hypertension, diabetes mellitus, and CKD. Since the retrosternal route was chosen in approximately half of the patients, intrathoracic, posterior mediastinal, and other routes were combined in the multivariable logistic regression analysis. On multivariable logistic regression analysis, the Brinkman index, hypertension, CKD, duration of anesthesia, epidural anesthesia, and RBC transfusion were significantly associated with AKI. During the process of backward stepwise selection, age, sex, BMI, diabetes mellitus, heart disease, peripheral vascular disease, ARBs, preoperative hemoglobin concentration, preoperative albumin concentration, dopamine, anesthetic agent, MIE, reconstruction route, total intravenous fluid volume, hydroxyethyl starch, and estimated blood loss were eliminated from the multivariable regression analysis model (Table 3). Of the anesthesia-related factors, only epidural anesthesia was significantly associated with AKI. Therefore, in the sensitivity analysis, the five factors other than epidural anesthesia were excluded one by one, and multivariable logistic regression analysis was performed. All models showed that epidural anesthesia was independently associated with AKI (Table 4).

**Table 3:** Multivariable logistic regression analysis for postoperative acute kidney injury

Variable	OR unadj	OR adj	95% CI	*P*-value
Age (y)	1.03	------	--------------	--------
Sex (male)	3.55	------	--------------	--------
Body mass index	1.08	------	--------------	--------
Brinkman index (per 100 units)	1.08	1.06	1.01—1.11	0.016
Hypertension	4.42	3.39	1.73 — 6.64	< 0.001
Diabetes mellitus	2.46	------	--------------	--------
Heart disease	3.10	------	--------------	--------
Chronic kidney disease	3.92	2.58	1.35 — 4.95	0.0042
Peripheral vascular disease	4.18	------	--------------	--------
ARB	2.23	------	--------------	--------
Preoperative hemoglobin concentration (g/dL)	0.84	------	--------------	--------
Preoperative albumin concentration (mg/dL)	0.44	------	--------------	--------
Administration of dopamine	2.31	------	--------------	--------
Duration of anesthesia (per 10 min)	1.04	1.03	1.00 — 1.06	0.032
Epidural anesthesia	0.25	0.35	0.17 — 0.74	0.0058
Anesthetic agent				
Inhalational	2.00	------	--------------	--------
TIVA	1 (reference)			
Surgery				
MIE	0.54	------	--------------	--------
Open thoracotomy	1 (reference)			
Reconstruction route				
Retrosternal route	0.71	------	--------------	--------
Other routes	1 (reference)			
Total intravenous fluid volume (per 100 mL)	1.02	------	--------------	--------
Hydroxyethyl starch (per 100 mL)	1.00	------	--------------	--------
Estimated blood loss (per 100 mL)	1.11	------	--------------	--------
RBC transfusion	4.00	3.27	1.18 — 9.08	0.023

*OR unadj* odds ratio unadjusted, *OR adj* odds ratio adjusted, *CI* confidence interval, *ARB* angiotensin II receptor blocker, *TIVA* total intravenous anesthesia, *MIE* minimally invasive esophagectomy, *RBC* red blood cell

**Table 4:** Sensitivity analysis

Variable	OR adj	95% CI	*P*-value
Model 1: Brinkman index eliminated from the original model			
Chronic kidney disease	2.65	1.39 — 5.06	0.0030
Duration of anesthesia (per 10 min)	1.03	1.01 — 1.06	0.016
Epidural anesthesia	0.35	0.17 — 0.73	0.0050
Hypertension	3.47	1.77 — 6.79	< 0.001
RBC transfusion	3.23	1.17 — 8.90	0.023
Model 2: Hypertension eliminated from the original model			
Brinkman index (per 100 units)	1.06	1.01 — 1.12	0.010
Chronic kidney disease	3.08	1.63 — 5.84	< 0.001
Duration of anesthesia (per 10 min)	1.03	1.00 — 1.05	0.043
Epidural anesthesia	0.32	0.15 — 0.66	0.0022
RBC transfusion	4.00	1.47 — 10.9	0.0066
Model 3: Chronic kidney disease eliminated from the original model			
Brinkman index (per 100 units)	1.06	1.01 — 1.12	0.011
Duration of anesthesia (per 10 min)	1.03	1.00 — 1.06	0.035
Epidural anesthesia	0.31	0.15 — 0.63	0.0014
Hypertension	3.78	1.94 — 7.35	< 0.001
RBC transfusion	3.62	1.35 — 9.74	0.011
Model 4: Duration of anesthesia eliminated from the original model			
Brinkman index (per 100 units)	1.07	1.02 — 1.12	0.0085
Chronic kidney disease	2.55	1.33 — 4.86	0.0046
Epidural anesthesia	0.32	0.15 — 0.66	0.0020
Hypertension	3.30	1.69 — 6.45	< 0.001
RBC transfusion	3.59	1.32 — 9.79	0.013
Model 5: RBC transfusion eliminated from the original model			
Brinkman index (per 100 units)	1.06	1.01 — 1.11	0.023
Chronic kidney disease	2.56	1.34 — 4.87	0.0044
Duration of anesthesia (per 10 min)	1.03	1.01 — 1.06	0.013
Epidural anesthesia	0.36	0.17 — 0.75	0.0059
Hypertension	3.61	1.84 — 7.05	< 0.001
Preoperative albumin concentration	0.43	0.19 — 0.97	0.041

*OR adj* odds ratio adjusted, *CI* confidence interval, *RBC* red blood cell

No significant difference in in-hospital mortality was found between AKI and non-AKI patients. There were significant differences in length of hospital stay and incidence of reintubation between AKI and non-AKI patients. The median length of hospital stay was 19 days, and the frequency of patients with a length of hospital stay exceeding 19 days was significantly higher in AKI patients than in non-AKI patients (Table 5).

**Table 5:** Outcomes

	AKI (*n* = 48)	Non-AKI (*n* = 957)	*P* value
In-hospital mortality (n (%))	1 (2.1)	7 (0.7)	0.32
Hospital stay (days, IQR)	26 (22-31)	18 (15-25)	<0.001
Hospital stay > 19 days (n (%))	40 (83.3)	426 (44.5)	<0.001
Re-intubation (n (%))	14 (29.2)	50 (5.2)	<0.001

*AKI* acute kidney injury, *IQR* interquartile range

## Discussion

The incidence of AKI within 72 h after esophagectomy and factors related to it were evaluated. The incidence of early postoperative AKI was 4.8%, with no significant difference between open thoracotomy and MIE. On multivariable logistic regression analysis, epidural anesthesia appeared to have a protective effect against early postoperative AKI, whereas the type of anesthetic agent, the total intravenous fluid volume, and the amount of hydroxyethyl starch were not associated with it. Although there was no difference in in-hospital mortality, the length of hospital stay was significantly longer in AKI patients than in non-AKI patients. 

Several studies have investigated early AKI after esophageal surgery [8, 13–15]; however, few have examined the relationship between AKI and factors related to anesthetic management, such as a comparison between inhaled anesthetics and TIVA, the effects of epidural anesthesia, the amount of intravenous fluid administered, and hydroxyethyl starch. For example, Wang et al. [8] analyzed epidural anesthesia as a factor related to anesthetic management in patients undergoing esophageal surgery, but other reports did not take into account the type of anesthetic agent or epidural anesthesia. In the study by Wang et al. [8], no difference was found in early postoperative AKI between patients who received epidural anesthesia and those who did not. The reason for the difference in the effect of epidural anesthesia on postoperative AKI between the study by Wang et al. [8] and the present study is unclear, but it may be related to the selection of epidural anesthesia patients between their study and the present one. In other words, we basically use epidural anesthesia as a means of analgesia in esophageal surgery, with a usage frequency of 91%, whereas Wang et al. used it only in a limited number of patients, with a usage frequency of 16% [8].

In the present study, the incidence of AKI within 72 h after esophagectomy was 4.8%, which is within the range reported in other studies (2.4–35.3%) [8, 13–15]. Furthermore, there was no difference in the incidence of AKI between open and thoracoscopic surgery (6.9 vs. 3.9%) in the present study. Some published studies have reported that thoracoscopic surgery is associated with a lower incidence of AKI than open surgery [2]. The mechanism underlying the difference in the incidence of postoperative AKI between MIE and open thoracotomy may be that open thoracotomy is more invasive and, therefore produces a stronger inflammatory response than MIE [16], since inflammation is thought to be one of the mechanisms of postoperative AKI [17]. The difference between the study by Dyas et al. [2] and the present study regarding whether there was a difference in the frequency of AKI between MIE and open thoracotomy may be related to the fact that the international definition of AKI was not used in the study by Dyas et al. [2]. However, the small number of patients in the present study, particularly those who underwent open thoracotomy, may have prevented the detection of a significant difference.

Multivariable logistic regression analysis in the present study showed that epidural anesthesia was significantly associated with a reduction in early postoperative AKI. To the best of our knowledge, no previous studies have demonstrated the effectiveness of epidural analgesia in reducing early postoperative AKI in patients undergoing esophagectomy. However, apart from esophagectomy, there are two randomized controlled trials that have investigated the effect of epidural anesthesia on postoperative renal dysfunction. In a study in which 188 pediatric patients undergoing cardiac surgery under cardiopulmonary bypass were randomly divided into two groups [18], one of which received epidural anesthesia and the other did not, the frequency of AKI requiring postoperative renal replacement therapy was shown to be significantly higher in the group that did not receive epidural anesthesia. In a study in which 40 patients undergoing robot-assisted urological surgery were randomly divided into two groups [19], one of which received combined general-epidural anesthesia and the other general anesthesia alone, there was no significant difference in the incidence of postoperative AKI, but postoperative plasma neutrophil gelatinase associated lipocalin concentrations were significantly lower in the combined general-epidural anesthesia group. Furthermore, epidural anesthesia has been shown to reduce postoperative AKI in abdominal aortic aneurysm repair [20]. Although the mechanism was unclear in these studies, it was assumed that the reduction in postoperative AKI by epidural anesthesia involves sympathetic nerve blocking effects, anti-inflammatory effects, and reduced cytokine production [19, 20]. Previous studies have shown the effectiveness of epidural anesthesia in reducing inflammatory and stress responses during and after esophageal surgery [21–23]. Although it is not possible to prove the involvement of inflammatory responses or stress responses, a similar mechanism to that described above may have acted to reduce AKI through epidural anesthesia in the present study. However, since the reason for not performing epidural anesthesia was not fully specified in the present study, the possibility that factors not measured in this study may have been involved cannot be excluded.

Various guidelines for esophageal surgery strongly recommend epidural analgesia as a method of postoperative analgesia [24, 25]. Epidural analgesia has been shown to contribute to reducing postoperative complications such as pneumonia and anastomotic leaks in patients after esophageal surgery [26], but its effectiveness in preventing postoperative AKI has not been established. The present study may suggest that one of the mechanisms by which postoperative epidural analgesia improves the outcomes of patients undergoing esophagectomy is its effect to prevent postoperative AKI.

A study by Wang et al. has already shown that smoking is significantly associated with AKI after esophageal cancer surgery [8]. Although smoking was independently associated with early postoperative AKI in the present study, it may not be clinically significant since the odds ratio per 100 units of the Brinkman index was small (1.06). The effect of smoking on postoperative AKI has not been fully investigated, but it is thought to involve endothelial dysfunction, activation of the renin-angiotensin system [27, 28], and oxidative stress [29]. Hypertension, CKD, and RBC transfusion, which were shown to be associated with AKI in the present study, have been established as risk factors for postoperative AKI [17]. The duration of anesthesia may reflect the complexity, difficulty, and invasiveness of the surgery.

Although no independent association was found between dopamine and postoperative AKI, the frequency of dopamine use was significantly higher in the AKI group. The reason for this is unclear, however, since one of the studies investigating the effects of dopamine on the kidney [30] showed that dopamine increased urinary retinol-binding protein (a biomarker of early renal tubular damage) in patients after coronary artery bypass surgery, the possibility that dopamine had a harmful effect on the kidney in this study cannot be excluded.

Several limitations of the present study need to be addressed. First, as with all observational research, residual or unmeasured confounders may be an alternative explanation for the results. Generalizability is limited to centers with patient and surgical profiles similar to our own. Second, intraoperative hypotension is an important risk factor for AKI in noncardiac surgery [31], but detailed intraoperative hemodynamic data were not available in this study. The lack of sufficient data on oxygenation during surgery is also considered to be one of the limitations of this study. Last, the number of patients in this study was relatively small, and it was necessary to include the entire cohort, precluding sample size calculation.

## Conclusions

The incidence of AKI within 72 h after surgery was 4.8%. Of the factors associated with anesthetic management, epidural anesthesia may have a protective effect against early postoperative AKI, whereas the type of anesthetic agent, total intravenous fluid volume, and the volume of hydroxyethyl starch infusion were not associated with early postoperative AKI. Epidural anesthesia should be encouraged in patients undergoing esophagectomy for esophageal cancer. Further research is needed to obtain clear results on the impact of MIE on postoperative AKI. Length of hospital stay was significantly longer in AKI patients than in non-AKI patients.

## Data Availability

The datasets used and/or analyzed during this study are available from the corresponding author on reasonable request.

## Abbreviations

MIE: Minimally invasive esophagectomy
AKI: Acute kidney injury
STROBE: Strengthening the Reporting of Observational Studies in Epidemiology
ASA-PS: American Society of Anesthesiologists physical status
CKD: Chronic kidney disease
UICC: Union for International Cancer Control
TNM: Tumor node metastasis
ACE: Angiotensin-converting enzyme
ARB: Angiotensin II receptor blocker
NSAID: Non-steroid anti-inflammatory drug
sCr: Serum creatinine
eGFR: Estimated glomerular filtration rate
TIVA: Total intravenous anesthesia
RBC: Red blood cell
KDIGO: Kidney Disease Improving Global Outcomes
VIF: Variance inflation factors

## References

[1] Oshikiri T, Takiguchi G, Miura S, Takase N, Hasegawa H, Yamamoto M, et al. Current status of minimally invasive esophagectomy for esophageal cancer: Is it truly less invasive? Ann Gastroenterol Surg. 2018;3:138–45. 30923783 10.1002/ags3.12224 PMC6422792

[2] Dyas AR, Stuart CM, Bronsert MR, Schulick RD, McCarter MD, Meguid RA. Minimally invasive surgery is associated with decreased postoperative complications after esophagectomy. J Thorac Cardiovasc Surg. 2023;166:268–78. 36577613 10.1016/j.jtcvs.2022.11.026

[3] Sakamoto T, Fujiogi M, Matsui H, Fushimi K, Yasunaga H. Comparing perioperative mortality and morbidity of minimally invasive esophagectomy versus open esophagectomy for esophageal cancer: A nationwide retrospective analysis. Ann Surg. 2021;274:324–30. 31356263 10.1097/SLA.0000000000003500

[4] Kim CS, Oak CY, Kim HY, Kang YU, Choi JS, Bae EH, et al. Incidence, predictive factors, and clinical outcomes of acute kidney injury after gastric surgery for gastric cancer. PLoS ONE. 2013;8:e82289. 24349249 10.1371/journal.pone.0082289 PMC3857284

[5] Tomozawa A, Ishikawa S, Shiota N, Cholvisudhi P, Makita K. Perioperative risk factors for acute kidney injury after liver resection surgery: an historical cohort study. Can J Anesth. 2015;62:753–61. 25925634 10.1007/s12630-015-0397-9

[6] Hansen MK, Gammelager H, Mikkelsen MM, Hjortdal VE, Layton JB, Johnsen SP, et al. Post-operative acute kidney injury and five-year risk of death, myocardial infarction, and stroke among elective cardiac surgical patients: a cohort study. Crit Care. 2013;17:R292. 24330762 10.1186/cc13158 PMC4057271

[7] Ishikawa S, Tanaka M, Maruyama F, Fukagawa A, Shiota N, Matsumura S, et al. Effects of acute kidney injury after liver resection on long-term outcomes. Korean J Anesthesiol. 2017;70:527–34. 29046772 10.4097/kjae.2017.70.5.527 PMC5645585

[8] Wang W, Wang T, Feng X, Sun L. Incidence and risk factors of acute kidney injury after esophageal cancer surgery: A nested case-control study. Int J Surg. 2017;39:11–5. 28104467 10.1016/j.ijsu.2017.01.043

[9] von Elm E, Altman DG, Egger M, Pocock SJ, Gøtzsche PC, Vandenbroucke JP, STROBE Initiative. The Strengthening the Reporting of Observational Studies in Epidemiology (STROBE) Statement: guidelines for reporting observational studies. Ann Intern Med. 2007;147:573–7. 17938396 10.7326/0003-4819-147-8-200710160-00010

[10] Matsuno S, Imai E, Horino M, Yasuda Y, Tomita K, Mitta K, et al. Collaborators developing the Japanese equation for estimated GFR. Revised equations for estimated GFR from serum creatinine in Japan. Am J Kidney Dis. 2009;53:982–92. 19339088 10.1053/j.ajkd.2008.12.034

[11] Khwaja A. KDIGO clinical practice guidelines for acute kidney injury. Nephron Clin Pract. 2012;120:c179–84. 10.1159/000339789 22890468

[12] Kanda Y. Investigation of the freely available easy-to-use software ‘EZR’ for medical statistics. Bone Marrow Transplant. 2013;48:452–8. 23208313 10.1038/bmt.2012.244 PMC3590441

[13] Lee EH, Kim HR, Baek SH, Kim KM, Chin JH, Choi DK, et al. Risk factors of postoperative acute kidney injury in patients undergoing esophageal cancer surgery. J Cardiothorac Vasc Anesth. 2014;28:936–42. 24680132 10.1053/j.jvca.2013.12.006

[14] Murphy CF, Dunne T, Elliott JA, Kamarajah SK, Leighton J, Evans RPT, et al. Acute kidney injury after esophageal cancer surgery: Incidence, risk factors, and impact on oncologic outcomes. Ann Surg. 2022;275:e683–9. 32740248 10.1097/SLA.0000000000004146

[15] Konda P, Ai D, Gurra CE, Rodriguez-Restrepo A, Mehran RJ, Rice D, et al. Identification of risk factors associated with postoperative acute kidney injury after esophagectomy for esophageal cancer. J Cardiothorac Vasc Anesth. 2017;31:474–81. 27720491 10.1053/j.jvca.2016.07.030

[16] Kanekiyo S, Takeda S, Tsutsui M, Nishiyama M, Kitahara M, Shindo Y, et al. Low invasiveness of thoracoscopic esophagectomy in the prone position for esophageal cancer: a propensity score-matched comparison of operative approaches between thoracoscopic and open esophagectomy. Surg Endosc. 2018;32:1945–53. 29075967 10.1007/s00464-017-5888-z

[17] Boyer N, Eldridge J, Prowle JR, Forni LG. Postoperative acute kidney injury. Clin J Am Soc Nephrol. 2022;17:1535–45. 35710717 10.2215/CJN.16541221 PMC9528271

[18] Kumar A, Ghotra GS, Dwivedi D, Bhargava DV, Joshi A, Tiwari N, et al. Common inflammatory markers and outcome after pediatric cardiac surgery with high thoracic epidural anesthesia: A randomized controlled study. World J Pediatr Congenit Heart Surg. 2023;14:334–44. 36823972 10.1177/21501351221151053

[19] Orsolya M, Attila-Zoltan M, Gherman V, Zaharie F, Bolboaca S, Chira C, et al. The effect of anaesthetic management on neutrophil gelatinase associated lipocalin (NGAL) level after robotic surgical oncology. JBUON. 2015;20:317–24. 25778333

[20] Salata K, Abdallah FW, Hussain MA, de Mestral C, Greco E, Aljabri B, et al. Short-term outcomes of combined neuraxial and general anaesthesia versus general anaesthesia alone for elective open abdominal aortic aneurysm repair: retrospective population-based cohort study. Br J Anaesth. 2020;124:544–52. 32216957 10.1016/j.bja.2020.01.018

[21] Fares KM, Mohamed SA, Hamza HM, Sayed DM, Hetta DF. Effect of thoracic epidural analgesia on proinflammatory cytokines in patients subjected to protective lung ventilation during Ivor Lewis esophagectomy. Pain Physician. 2014;17:305–15. 25054390

[22] Wang J, Yin Y, Zhu Y, Xu P, Sun Z, Miao C, et al. Thoracic epidural anaesthesia and analgesia ameliorates surgery-induced stress response and postoperative pain in patients undergoing radical oesophagectomy. J Int Med Res. 2019;47:6160–70. 31426685 10.1177/0300060519866943 PMC7045687

[23] Gu CY, Zhang J, Qian YN, Tang QF. Effects of epidural anesthesia and postoperative epidural analgesia on immune function in esophageal carcinoma patients undergoing thoracic surgery. Mol Clin Oncol. 2015;3:190–6. 25469293 10.3892/mco.2014.405 PMC4251267

[24] Findlay JM, Gillies RS, Millo J, Sgromo B, Marshall RE, Maynard ND. Enhanced recovery for esophagectomy: a systematic review and evidence-based guidelines. Ann Surg. 2014;259:413–31. 24253135 10.1097/SLA.0000000000000349

[25] Low DE, Allum W, De Manzoni G, Ferri L, Immanuel A, Kuppusamy M, et al. Guidelines for perioperative care in esophagectomy: Enhanced Recovery After Surgery (ERAS(®)) Society recommendations. World J Surg. 2019;43:299–330. 30276441 10.1007/s00268-018-4786-4

[26] Li W, Li Y, Huang Q, Ye S, Rong T. Short and long-term outcomes of epidural or intravenous analgesia after esophagectomy: A propensity-matched cohort study. PLoS ONE. 2016;11:e0154380. 27110939 10.1371/journal.pone.0154380 PMC4844138

[27] Orth SR. Effects of smoking on systemic and intrarenal hemodynamics: influence on renal function. J Am Soc Nephrol. 2004;15(Suppl 1):S58–63. 14684675 10.1097/01.asn.0000093461.36097.d5

[28] Orth SR. Smoking and the kidney. J Am Soc Nephrol. 2002;13:1663–72. 12039997 10.1097/01.asn.0000018401.82863.fd

[29] Arany I, Grifoni S, Clark JS, Csongradi E, Marik C, Juncos LA. Chronic nicotine exposure exacerbates acute renal ischemic injury. Am J Physiol Renal Physiol. 2011;301:F125–33. 21511693 10.1152/ajprenal.00041.2011 PMC3129886

[30] Tang AT, El-Gamel A, Keevil B, Yonan N, Deiraniya AK. The effect of “renal-dose” dopamine on renal tubular function following cardiac surgery: assessed by measuring retinol binding protein (RBP). Eur J Cardiothorac Surg. 1999;15:717–21. 10386423 10.1016/s1010-7940(99)00081-0

[31] Walsh M, Devereaux PJ, Garg AX, Kurz A, Turan A, Rodseth RN, et al. Relationship between intraoperative mean arterial pressure and clinical outcomes after noncardiac surgery: toward an empirical definition of hypotension. Anesthesiology. 2013;119:507–15. 23835589 10.1097/ALN.0b013e3182a10e26

